# Supplementary material for: Prevalence and Antifungal Susceptibility of Clinically Relevant Candida Species, Identification of Candida auris and Kodamaea ohmeri in Bangladesh
Source: Trop Med Infect Dis. 2022 Aug 26;7(9):211. doi: 10.3390/tropicalmed7090211 (PMC9506023; doi:10.3390/tropicalmed7090211)
Supplement: Supplementary file 1 [file tropicalmed-07-00211-s001.zip › supplementary-Figure S3.pdf]

(a)

```
A173-22       ---------------ACTGTCATTGATGGCATTAATTATTTTTTGTCCCTTAGTGTTACA    45
A1201-57      ---------------ACTGTCATTGATGGCATTAATTATTTTTTGTCCCTTAGTGTTACA    45
SC5314        ATGGCTATTGTTGAAACTGTCATTGATGGCATTAATTATTTTTTGTCCCTTAGTGTTACA    60
                             *********************************************

A173-22       CAACAGATCAGTATATTATTAGGGGTTCCATTTGTTTACAACTTAGTATGGCAATATTTA    105
A1201-57      CAACAGATCAGTATATTATTAGGGGTTCCATTTGTTTACAACTTAGTATGGCAATATTTA    105
SC5314        CAACAGATCAGTATATTATTAGGGGTTCCATTTGTTTACAACTTAGTATGGCAATATTTA    120
              ************************************************************

A173-22       TATTCATTAAGAAAAGATAGAGCTCCATTAGTGTTTTATTGGATTCCTTGGTTTGGTTCT    165
A1201-57      TATTCATTAAGAAAAGATAGAGCTCCATTAGTGTTTTATTGGATTCCTTGGTTTGGTTCT    165
SC5314        TATTCATTAAGAAAAGATAGAGCTCCATTAGTGTTTTATTGGATTCCTTGGTTTGGTTCT    180
              ************************************************************

A173-22       GCAGCTTCATATGGTCAACAACCTTATGAATTTTTCGAATCATGTCGTCAAAAGTATGGT    225
A1201-57      GCAGCTTCATATGGTCAACAACCTTATGAATTTTTCGAATCATGTCGTCAAAAGTATGGT    225
SC5314        GCAGCTTCATATGGTCAACAACCTTATGAATTTTTCGAATCATGTCGTCAAAAGTATGGT    240
              ************************************************************

A173-22       GATGTATTTTCATTTATGTTATTAGGGAAAATTATGACGGTTTATTTAGGTCCAAAAGGT    285
A1201-57      GATGTATTTTCATTTATGTTATTAGGGAAAATTATGACGGTTTATTTAGGTCCAAAAGGT    285
SC5314        GATGTATTTTCATTTATGTTATTAGGGAAAATTATGACGGTTTATTTAGGTCCAAAAGGT    300
              ************************************************************

A173-22       CATGAATTTGTTTTTAATGCTAAATTATCTGATGTTTCTGTTGAAGATGCTTATAAACAT    345
A1201-57      CATGAATTTGTTTTTAATGCTAAATTATCTGATGTTTCTGTTGAAGATGCTTATAAACAT    345
SC5314        CATGAATTTGTTTTTAATGCTAAATTATCTGATGTTTCTGCTGAAGATGCTTATAAACAT    360
              **************************************** *******************
                                                     341

A173-22       TTAACTACTCCAGTTTTCGGTAAAGGGGTTATTTATGATTGTCCAAATTCCAGATTAATG    405
A1201-57      TTAACTACTCCAGTTTTCGGTAAAGGGGTTATTTATGATTGTCCAAATTCCAGATTAATG    405
SC5314        TTAACTACTCCAGTTTTCGGTAAAGGGGTTATTTATGATTGTCCAAATTCCAGATTAATG    420
              ************************************************************

A173-22       GAACAAAAAAAACTTGCTAAATTTGCTTTGACTACTGATTCATTTAAAAGATATGTTCCT    465
A1201-57      GAACAAAAAAAATTAGCTAAATTTGCTTTGACTACTGATTCATTTAAAAGATATGTTCCT    465
SC5314        GAACAAAAAAAATTTGCTAAATTTGCTTTGACTACTGATTCATTTAAAAGATATGTTCCT    480
              ************ * *********************************************
                          433,435

A173-22       AAGATTAGAGAAGAAATTTTGAATTATTTTGTTACTGATGAAAGTTTCAAATTGAAAGAA    525
A1201-57      AAGATTAGAGAAGAAATTTTGAATTATTTTGTTACTGATGAAAGTTTCAAATTGAAAGAA    525
SC5314        AAGATTAGAGAAGAAATTTTGAATTATTTTGTTACTGATGAAAGTTTCAAATTGAAAGAA    540
              ************************************************************

A173-22       AAAACTCATGGGGTTGCCAATGTTATGAAAACTCAACCAGAAATTACTATTTTCACTGCT    585
A1201-57      AAAACTCATGGGGTTGCCAATGTTATGAAAACTCAACCAGAAATTACTATTTTCACTGCT    585
SC5314        AAAACTCATGGGGTTGCCAATGTTATGAAAACTCAACCAGAAATTACTATTTTCACTGCT    600
              ************************************************************

A173-22       TCAAGATCTTTATTTGGTGATGAAATGAGAAGAATTTTTTGACCGTTCATTTGCTCAACTA    645
A1201-57      TCAAGATCTTTATTTGGTGATGAAATGAGAAGAATTTTTTGACCGTTCATTTGCTCAACTA    645
SC5314        TCAAGATCTTTATTTGGTGATGAAATGAGAAGAATTTTTTGACCGTTCATTTGCTCAACTA    660
              ************************************************************

A173-22       TATTCTGATTTAGATAAAGGTTTTACCCCTATTAATTTTGTTTTCCCTAATTTACCTTTA    705
A1201-57      TATTCTGATTTAGATAAAGGTTTTACCCCTATTAATTTTGTTTTCCCTAATTTACCTTTA    705
SC5314        TATTCTGATTTAGATAAAGGTTTTACCCCTATTAATTTTGTTTTCCCTAATTTACCTTTA    720
              ************************************************************

A173-22       CCTCATTATTGGAGACGTGATGCTGCTCAAAAGAAAATCTCTGCTACTTATATGAAAGAA    765
A1201-57      CCTCATTATTGGAGACGTGATGCTGCTCAAAAGAAAATCTCTGCTACTTATATGAAAGAA    765
SC5314        CCTCATTATTGGAGACGTGATGCTGCTCAAAAGAAAATCTCTGCTACTTATATGAAAGAA    780
              ************************************************************

A173-22       ATTAAACTGAGAAGAGAACGTGGTGATATTGATCCAAATCGTGATGTAATTGATTCCTTA    825
A1201-57      ATTAAACTGAGAAGAGAACGTGGTGATATTGATCCAAATCGTGATTTAATTGATTCCTTA    825
SC5314        ATTAAACTGAGAAGAGAACGTGGTGATATTGATCCAAATCGTGATTTAATTGATTCCTTA    840
              ********************************************* **************
                                                            826
```

```
A173-22     TTGATTCATTCAACTTATAAAGATGGTGTGAAAATGACTGATCAAGAAATTGCTAATCTT     885
A1201-57    TTGATTCATTCAACTTATAAAGATGGTGTGAAAATGACTGATCAAGAAATTGCTAATCTT     885
SC5314      TTGATTCATTCAACTTATAAAGATGGTGTGAAAATGACTGATCAAGAAATTGCTAATCTT     900
            ************************************************************

A173-22     TTAATTGGTATTCTTATGGGTGGTCAACATACTTCTGCTTCTACTTCTGCTTGGTTCTTG     945
A1201-57    TTAATTGGTATTCTTATGGGTGGTCAACATACTTCTGCTTCTACTTCTGCTTGGTTCTTG     945
SC5314      TTAATTGGTATTCTTATGGGTGGTCAACATACTTCTGCTTCTACTTCTGCTTGGTTCTTG     960
            ************************************************************

A173-22     TTACATTTAGGTGAAAAACCTCATTTACAAGATGTTATTTATCAAGAAGTTGTTGAATTA     1005
A1201-57    TTACATTTAGGTGAAAAACCTCATTTACAAGATGTTATTTATCAAGAAGTTGTTGAATTA     1005
SC5314      TTACATTTAGGTGAAAAACCTCATTTACAAGATGTTATTTATCAAGAAGTTGTTGAATTA     1020
            ************************************************************

A173-22     TTGAAAGAAAAAGGTGGTGATTTGAATGATTTGACTTATGAAGATTTACAAAAATTACCA     1065
A1201-57    TTGAAAGAAAAAGGTGGTGATTTGAATGATTTGACTTATGAAGATTTACAAAAATTACCA     1065
SC5314      TTGAAAGAAAAAGGTGGTGATTTGAATGATTTGACTTATGAAGATTTACAAAAATTACCA     1080
            ************************************************************

A173-22     TCAGTCAATAACACTATTAAGGAAACTCTCAGAATGCATATGCCATTACATTCTATTTTT     1125
A1201-57    TCAGTCAATAACACTATTAAGGAAACTCTCAGAATGCATATGCCATTACATTCTATTTTT     1125
SC5314      TCAGTCAATAACACTATTAAGGAAACTCTCAGAATGCATATGCCATTACATTCTATTTTT     1140
            ************************************************************

A173-22     AGAAAAGTTACTAACCCATTAAGAATCCCTGAAACCAATTATATTGTTCCAAAAGGTCAT     1185
A1201-57    AGAAAAGTTACTAACCCATTAAGAATCCCTGAAACCAATTATATTGTTCCAAAAGGTCAT     1185
SC5314      AGAAAAGTTACTAACCCATTAAGAATCCCTGAAACCAATTATATTGTTCCAAAAGGTCAT     1200
            ************************************************************

A173-22     TATGTTTTAGTTTCTCCAGGTTATGCTCATACTAGTGAAAGATATTTTGATAACCCTGAA     1245
A1201-57    TATGTTTTAGTTTCTCCAGGTTATGCTCATACTAGTGAAAGATATTTTGATAACCCTGAA     1245
SC5314      TATGTTTTAGTTTCTCCAGGTTATGCTCATACTAGTGAAAGATATTTTGATAACCCTGAA     1260
            ************************************************************

A173-22     GATTTTGATCCAACTAGATGGGATACTGCTGCTGCCAAAGCTAATTCTGTTTCATTTAAC     1305
A1201-57    GATTTTGATCCAACTAGATGGGATACTGCTGCTGCCAAAGCTAATTCTGTTTCATTTAAC     1305
SC5314      GATTTTGATCCAACTAGATGGGATACTGCTGCTGCCAAAGCTAATTCTGTTTCATTTAAC     1320
            ************************************************************

A173-22     TCTTCTGATGAAGTTGATTATGGGTTTGGGAAAGTTTCTAAAGGGGTTTCTTCACCTTAT     1365
A1201-57    TCTTCTGATGAAGTTGATTATGGGTTTGGGAAAGTTTCTAAAGGGGTTTCTTCACCTTAT     1365
SC5314      TCTTCTGATGAAGTTGATTATGGGTTTGGGAAAGTTTCTAAAGGGGTTTCTTCACCTTAT     1380
            ************************************************************

A173-22     TTACCATTTGGTGGTGGTAGACATAGATGTATTGGGGAACAATTTGCTTATGTTCAATTA     1425
A1201-57    TTACCATTTGGTGGTGGTAGACATAGATGTATTGGGGAACAATTTGCTTATGTTCAATTA     1425
SC5314      TTACCATTTGGTGGTGGTAGACATAGATGTATTGGGGAACAATTTGCTTATGTTCAATTA     1440
            ************************************************************

A173-22     GGAACCATTTTAACTACTTTTGTTTATAATTTAAGATGGACTATTGATGGTTATAAAGTG     1485
A1201-57    GGAACCATTTTAACTACTTTTGTTTATAATTTAAGATGGACTATTGATGGTTATAAAGTG     1485
SC5314      GGAACCATTTTAACTACTTTTGTTTATAATTTAAGATGGACTATTGATGGTTATAAAGTG     1500
            ************************************************************

A173-22     CCTGACCCTGATTATAGTTCAATGGTGGTTTTACCTACTGAACCAGCAGAAATCATTTGG     1545
A1201-57    CCTGACCCTGATTATAGTTCAATGGTGGTTTTACCTACTGAACCAGCAGAAATCATTTGG     1545
SC5314      CCTGACCCTGATTATAGTTCAATGGTGGTTTTACCTACTGAACCAGCAGAAATCATTTGG     1560
            ************************************************************

A173-22     GAAAAAAGAGAAACT------------          1560
A1201-57    GAAAAAAGAGAAACT------------          1560
SC5314      GAAAAAAGAGAAACTTGTATGTTTTAA          1587
            ***************
```

(b)

```
SC5314          MAIVETVIDGINYFLSLSVTQQISILLGVPFVYNLVWQYLYSLRKDRAPLVFYWIPWFGS     60
A173-22         -----TVIDGINYFLSLSVTQQISILLGVPFVYNLVWQYLYSLRKDRAPLVFYWIPWFGS     55
A1201-57        -----TVIDGINYFLSLSVTQQISILLGVPFVYNLVWQYLYSLRKDRAPLVFYWIPWFGS     55
                     *******************************************************

SC5314          AASYGQQPYEFFESCRQKYGDVFSFMLLGKIMTVYLGPKGHEFVFNAKLSDVSAEDAYKH     120
A173-22         AASYGQQPYEFFESCRQKYGDVFSFMLLGKIMTVYLGPKGHEFVFNAKLSDVSVEDAYKH     115
A1201-57        AASYGQQPYEFFESCRQKYGDVFSFMLLGKIMTVYLGPKGHEFVFNAKLSDVSVEDAYKH     115
                *****************************************************.******
                                                                     114

SC5314          LTTPVFGKGVIYDCPNSRLMEQKKFAKFALTTDSFKRYVPKIREEILNYFVTDESFKLKE     180
A173-22         LTTPVFGKGVIYDCPNSRLMEQKKLAKFALTTDSFKRYVPKIREEILNYFVTDESFKLKE     175
A1201-57        LTTPVFGKGVIYDCPNSRLMEQKKLAKFALTTDSFKRYVPKIREEILNYFVTDESFKLKE     175
                ************************:***********************************
                                        145

SC5314          KTHGVANVMKTQPEITIFTASRSLFGDEMRRIFDRSFAQLYSDLDKGFTPINFVFPNLPL     240
A173-22         KTHGVANVMKTQPEITIFTASRSLFGDEMRRIFDRSFAQLYSDLDKGFTPINFVFPNLPL     235
A1201-57        KTHGVANVMKTQPEITIFTASRSLFGDEMRRIFDRSFAQLYSDLDKGFTPINFVFPNLPL     235
                ************************************************************

SC5314          PHYWRRDAAQKKISATYMKEIKLRRERGDIDPNRDLIDSLLIHSTYKDGVKMTDQEIANL     300
A173-22         PHYWRRDAAQKKISATYMKEIKLRRERGDIDPNRDVIDSLLIHSTYKDGVKMTDQEIANL     295
A1201-57        PHYWRRDAAQKKISATYMKEIKLRRERGDIDPNRDLIDSLLIHSTYKDGVKMTDQEIANL     295
                ***********************************:************************
                                                   276

SC5314          LIGILMGGQHTSASTSAWFLLHLGEKPHLQDVIYQEVVELLKEKGGDLNDLTYEDLQKLP     360
A173-22         LIGILMGGQHTSASTSAWFLLHLGEKPHLQDVIYQEVVELLKEKGGDLNDLTYEDLQKLP     355
A1201-57        LIGILMGGQHTSASTSAWFLLHLGEKPHLQDVIYQEVVELLKEKGGDLNDLTYEDLQKLP     355
                ************************************************************

SC5314          SVNNTIKETLRMHMPLHSIFRKVTNPLRIPETNYIVPKGHYVLVSPGYAHTSERYFDNPE     420
A173-22         SVNNTIKETLRMHMPLHSIFRKVTNPLRIPETNYIVPKGHYVLVSPGYAHTSERYFDNPE     415
A1201-57        SVNNTIKETLRMHMPLHSIFRKVTNPLRIPETNYIVPKGHYVLVSPGYAHTSERYFDNPE     415
                ************************************************************

SC5314          DFDPTRWDTAAAKANSVSFNSSDEVDYGFGKVSKGVSSPYLPFGGGRHRCIGEQFAYVQL     480
A173-22         DFDPTRWDTAAAKANSVSFNSSDEVDYGFGKVSKGVSSPYLPFGGGRHRCIGEQFAYVQL     475
A1201-57        DFDPTRWDTAAAKANSVSFNSSDEVDYGFGKVSKGVSSPYLPFGGGRHRCIGEQFAYVQL     475
                ************************************************************

SC5314          GTILTTFVYNLRWTIDGYKVPDPDYSSMVVLPTEPAEIIWEKRETCMF         528
A173-22         GTILTTFVYNLRWTIDGYKVPDPDYSSMVVLPTEPAEIIWEKRET---         520
A1201-57        GTILTTFVYNLRWTIDGYKVPDPDYSSMVVLPTEPAEIIWEKRET---         520
                *********************************************
```

Figure S3. Alignment of nucleotide sequence (a) and amino acid sequence (b) of ERG11 gene/protein product of *C. albicans* isolates A173-22, A1201-57, and reference strain SC5314 (GenBank accession no. CP01762). Asterisk indicates identical nucleotide/amino acid among three strains. Nucleotide/amino acid which is different from that of the reference strain is shown in yellow with its number below the alignment.
