# Supplementary material for: Prevalence and Antifungal Susceptibility of Clinically Relevant Candida Species, Identification of Candida auris and Kodamaea ohmeri in Bangladesh
Source: Trop Med Infect Dis. 2022 Aug 26;7(9):211. doi: 10.3390/tropicalmed7090211 (PMC9506023; doi:10.3390/tropicalmed7090211)
Supplement: Supplementary file 1 [file tropicalmed-07-00211-s001.zip › supplementary-Figures S1 and S2.pdf]

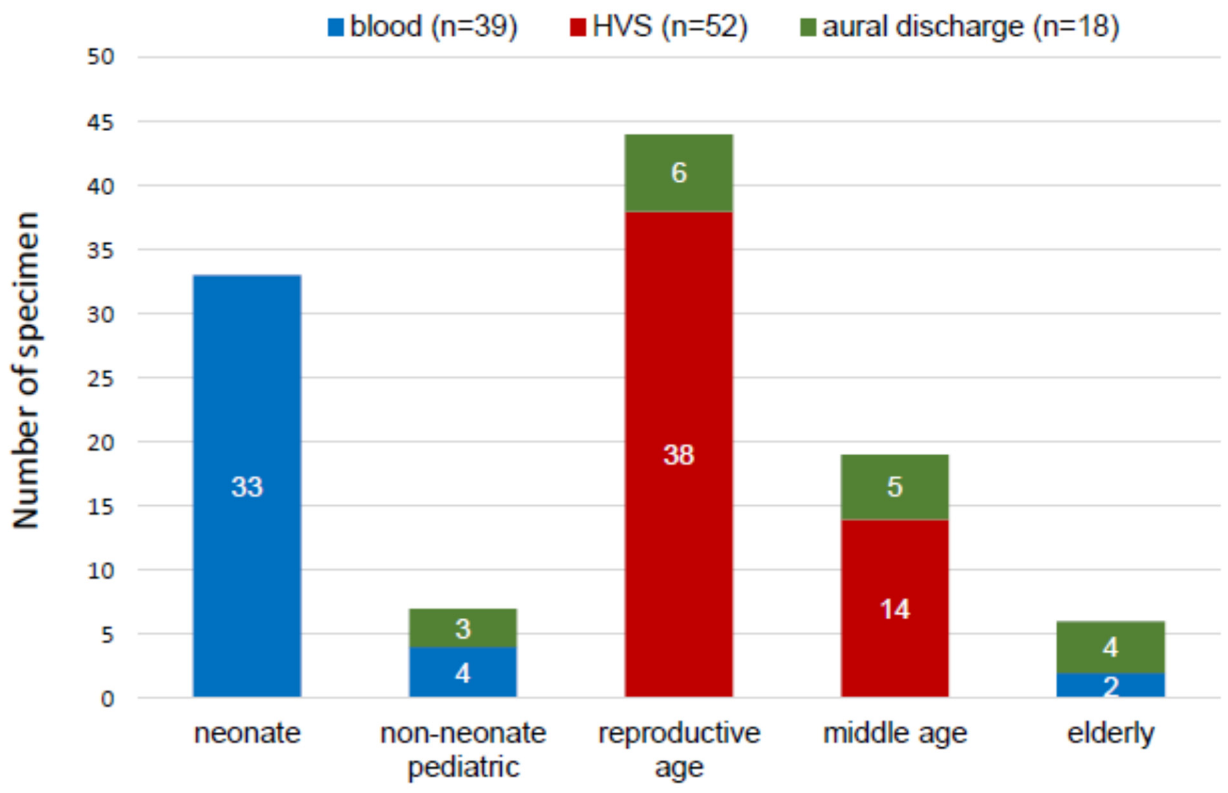

Figure S1. Frequency of candidiasis patients (specimen types) in each age group. Neonate, 0-28 days; non-neonate pediatric, 29 days-<18 years; reproductive age, 18-<45 years; middle age, 45-<60 years; elderly, ≥65 years. .

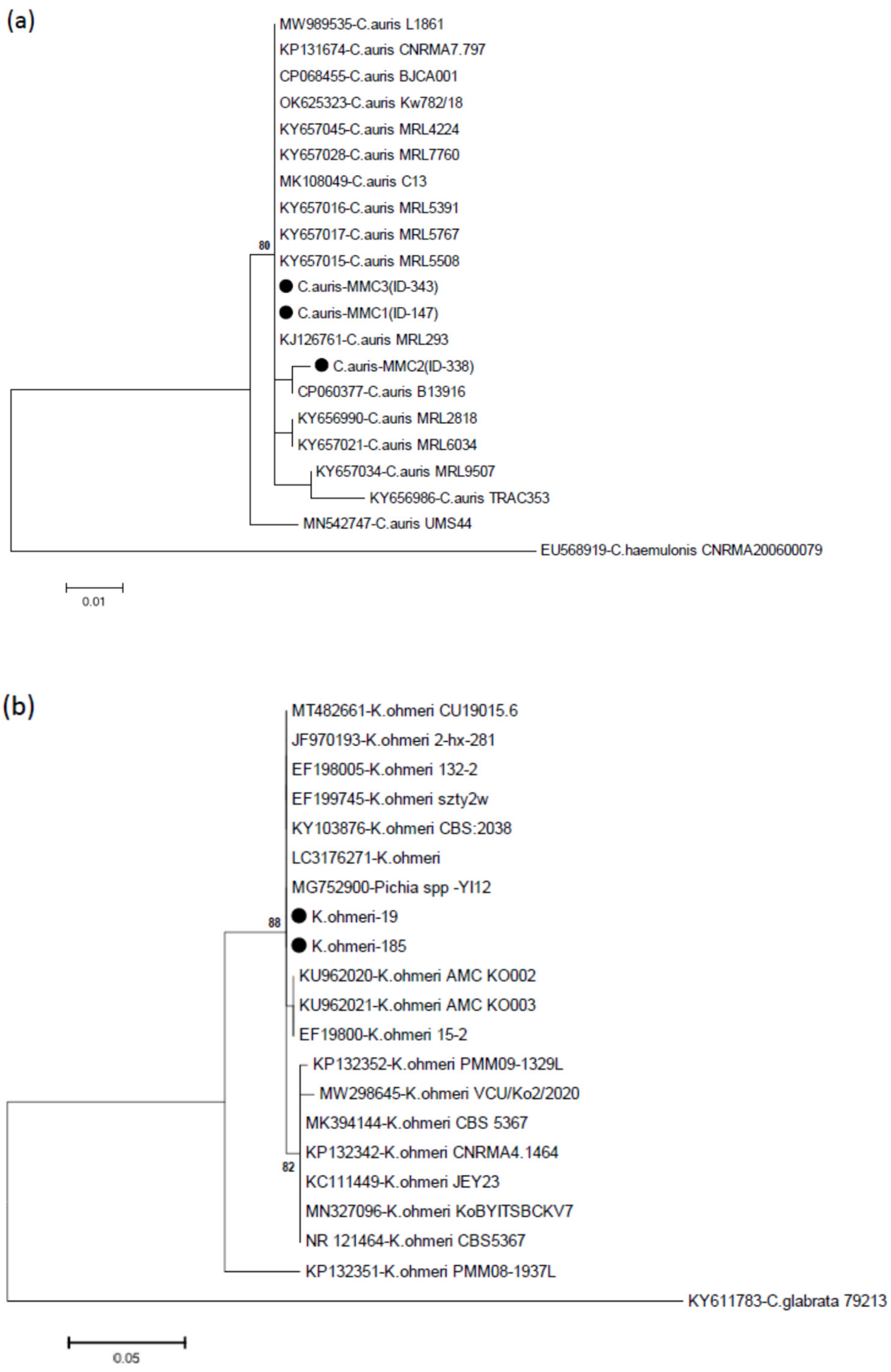

Figure S2. Phylogenetic dendrogram of ITS1-5.8S-ITS2 ribosomal region of the *C. auris* (a) and *K. ohmeri* (b) constructed by maximum-likelihood method with MEGA.11 program. Trees were statistically supported by bootstrapping with 1000 replicates, and genetic distances were calculated by Kimura two-parameter model. Variation scale is described at the bottom. Percent bootstrap support is indicated by the values at each node (the values < 80 are omitted). Filled circles indicate isolates analyzed in the present study. Other sequences were retrieved from GenBank database. Sequences of *C.haemulonis* CNRMA200600079 and *C. glabrata* 79213 were added as outgroup for *C. auris* and *K. ohmeri* , respectively.
