# Supplementary material for: Prevalence and Antifungal Susceptibility of Clinically Relevant Candida Species, Identification of Candida auris and Kodamaea ohmeri in Bangladesh
Source: Trop Med Infect Dis. 2022 Aug 26;7(9):211. doi: 10.3390/tropicalmed7090211 (PMC9506023; doi:10.3390/tropicalmed7090211)
Supplement: Supplementary file 1 [file tropicalmed-07-00211-s001.zip › supplementary-Tables S1-S3.pdf]

Table S1. Isolate numbers of each *Candida* species identified by different methods.

Candida Species	Number of Isolates	Identification Method					
		Germ Tube Test	Chromogenic Agar Media	PCR	PCR followed by RFLP	Citrate Utilization Test*1	Sequence Analysis*2
C. albicans	40	38	37	37	40	-	-
C. parapsilosis	25	0	4	20	25	-	-
C. ciferrii	12	0	0	8	8	4	-
C. tropicalis	10	0	8	3	10	-	-
C. glabrata	6	0	3	6	6	-	-
C. rugosa	4	0	0	2	4	-	-
C. famata	3	0	0	3	3	-	-
C. auris	3	0	0	0	0	0	3
K. ohmeri	2	0	0	0	0	0	2
C. krusei	1	0	1	1	1	-	-
C. kefyr	1	0	0	1	1	-	-
C. lusitaniae	1	0	0	1	1	-	-
C. dubliniensis	1	1	1	0	1	-	-
Total	109	39	54	82	100	4	5

*1 This test was done for only for isolates of which the identification by PCR-RFLP showed ambiguous results.

*2 Sequence of ITS1-5.8S-ITS2 ribosomal region was analyzed for only 5 isolates that were not identified by other methods.

Table S2. MIC of Fluconazole against isolated *Candida* species by broth microdilution method (n = 109).

Candida species	No. of Isolates	MIC (µg/mL)							
		0.5	1	2	4	8	16	32	64
C. albicans	40	12	6	8	10	0	1	1	2
C. parapsilosis	25	0	5	12	5	0	0	0	3
C. ciferrii	12	0	0	2	2	0	0	0	8
C. tropicalis	10	0	2	3	4	0	0	1	0
C. glabrata	6	0	0	0	3	0	0	1	2
C. rugosa	4	0	0	0	4	0	0	0	0
C. famata	3	0	0	3	0	0	0	0	0
C. auris	3	0	0	0	0	0	0	0	3
K. ohmeri	2	0	0	0	0	0	0	2	0
C. krusei	1	0	0	0	0	0	0	1	0
C. kefyr	1	0	0	0	0	0	0	1	0
C. lusitaniae	1	0	1	0	0	0	0	0	0
C. dubliniensis	1	0	1	0	0	0	0	0	0

C. krusei is intrinsically resistant to fluconazole.

Table S3. MIC of Amphotericin B against of *Candida* species isolated from blood using the broth microdilution method (n = 39).

Candida species	No. of isolates	MIC (µg/mL)							
		0.0032	0.0063	0.125	0.25	0.5	1	2	4
C. albicans	4	0	0	0	2	2	0	0	0
C. parapsilosis	16	0	0	0	9	7	0	0	0
C. ciferrii	9	0	0	0	3	2	1	0	3
C. rugosa	4	0	0	0	1	3	0	0	0
C. auris	3	0	0	0	0	0	0	0	3
C. tropicalis	1	0	0	1	0	0	0	0	0
C. glabrata	1	0	0	0	1	0	0	0	0
C. lusitaniae	1	0	0	0	0	0	0	0	1
